# A monolithic microfluidic probe for ambient mass spectrometry imaging of biological tissues†

**DOI:** 10.1039/d3lc00637a

**Published:** 2023-09-25

**Authors:** Li-Xue Jiang, Matthias Polack, Xiangtang Li, Manxi Yang, Detlev Belder, Julia Laskin

**Affiliations:** a Department of Chemistry, Purdue University West Lafayette IN 47907 USA jlaskin@purdue.edu; b Institute of Analytical Chemistry, Leipzig University Leipzig 04103 Germany belder@uni-leipzig.de

## Abstract

Ambient mass spectrometry imaging (MSI) is a powerful technique that allows for the simultaneous mapping of hundreds of molecules in biological samples under atmospheric conditions, requiring minimal sample preparation. We have developed nanospray desorption electrospray ionization (nano-DESI), a liquid extraction-based ambient ionization technique, which has proven to be sensitive and capable of achieving high spatial resolution. We have previously described an integrated microfluidic probe, which simplifies the nano-DESI setup, but is quite difficult to fabricate. Herein, we introduce a facile and scalable strategy for fabricating microfluidic devices for nano-DESI MSI applications. Our approach involves the use of selective laser-assisted etching (SLE) of fused silica to create a monolithic microfluidic probe (SLE-MFP). Unlike the traditional photolithography-based fabrication, SLE eliminates the need for the wafer bonding process and allows for automated, scalable fabrication of the probe. The chamfered design of the sampling port and ESI emitter significantly reduces the amount of polishing required to fine-tune the probe thereby streamlining and simplifying the fabrication process. We have also examined the performance of a V-shaped probe, in which only the sampling port is fabricated using SLE technology. The V-shaped design of the probe is easy to fabricate and provides an opportunity to independently optimize the size and shape of the electrospray emitter. We have evaluated the performance of SLE-MFP by imaging mouse tissue sections. Our results demonstrate that SLE technology enables the fabrication of robust monolithic microfluidic probes for MSI experiments. This development expands the capabilities of nano-DESI MSI and makes the technique more accessible to the broader scientific community.

## Abstract 2

Novel laser-assisted etching of a fused silica microfluidic probe for liquid extraction-based ambient mass spectrometry imaging.

## Graphical abstract

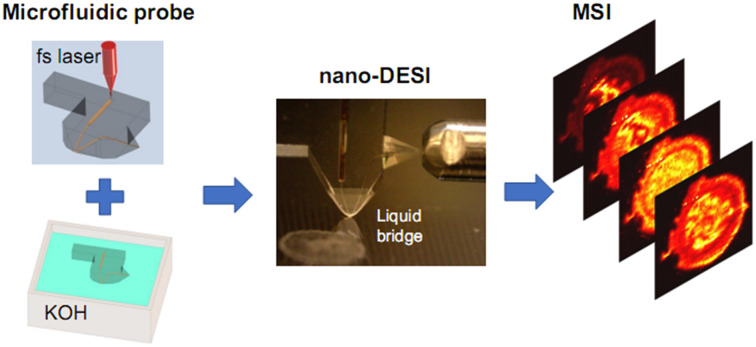

## 1. Introduction

Mass spectrometry imaging (MSI) is a powerful label-free method for mapping of multiple classes of biomolecules, including metabolites, fatty acids, lipids, peptides, and proteins in biological samples.^1–6^ Several ambient ionization-based MSI approaches have been developed for imaging of samples under atmospheric conditions with minimal sample preparation.^7–10^ These methods can be divided into two groups: techniques that rely on laser desorption/ablation of molecules and techniques that use localized liquid extraction to desorb analytes from the sample surface.^11^ Laser-based ambient MSI techniques include laser ablation electrospray ionization (LAESI)^12^ and infrared matrix-assisted laser desorption electrospray ionization (IR-MALDESI).^13^ Liquid extraction-based MSI techniques include desorption electrospray ionization (DESI),^14–18^ nanospray desorption electrospray ionization (nano-DESI),^19–22^ single probe,^23^ and tapping-mode scanning probe electrospray ionization (t-SPESI).^24^

Nano-DESI is a sensitive liquid extraction-based ionization technique, in which analytes are extracted from a sample into a dynamic liquid bridge formed between two fused-silica capillaries and ionized at a mass spectrometer inlet using electrospray ionization.^25^ Nano-DESI has been used for quantitative imaging of lipids,^26,27^ drugs,^28^ and metabolites,^20,29^ imaging of proteoforms^30–32^ and protein complexes,^19,33,34^ isomer-selective imaging of lipids,^35,36^ spatial profiling and imaging of microbial colonies.^37–39^ High spatial resolution of 10 μm has been achieved in nano-DESI MSI by finely pulling the two capillaries and incorporating a shear force probe.^40,41^ Recently, an integrated microfluidic probe (iMFP) has been developed to simplify the nano-DESI setup.^42^ The iMFP comprises a sampling port formed by two microfluidic channels that generate a liquid bridge on the sample surface. The iMFP simplifies the alignment process and enables high-throughput nano-DESI MSI experiments.^43^ Improving the throughput is critical for high spatial resolution MSI experiments.^44^ The second-generation iMFP with a redesigned sampling port has enabled imaging of large tissue sections with a spatial resolution of 10 μm.^45^ Despite these advantages of the iMFP, the manufacturing process is complex, costly, and requires sophisticated equipment and cleanroom facilities for photolithography, chemical etching, and high temperature glass–glass fusion bonding.

To reduce technical barriers for fabricating microfluidic devices for nano-DESI MSI, we have developed a facile and scalable fabrication strategy using selective laser-induced etching (SLE) technology.^46,47^ The SLE process uses a tightly focused femtosecond IR-laser beam that triggers non-linear absorption phenomena in glass material.^48,49^ By carefully selecting laser and process parameters, local material modifications are introduced in the vicinity of the laser focal point. This enables the inscription of complex geometries and channels inside fused silica by sweeping the IR-laser beam along a predetermined path through the material. The laser-modified areas are then selectively removed using an etching solution that exhibits a significantly higher etch rate and selectivity towards the laser-modified regions compared to pristine fused silica.^50,51^ In contrast to conventional photolithography, SLE manufacturing allows for the creation of custom free-form three-dimensional structures that otherwise would be impractical to achieve.^52–55^ This eliminates time-consuming manual steps such as spin coating of photoresist, structuring *via* optical masks, or error-prone glass–glass bonding. In addition, the SLE process does not require a particle-free environment, which further reduces the need for sophisticated equipment and cleanroom facilities. SLE technology has already demonstrated its effectiveness at fabricating microfluidic devices for applications in separations, photonics, and sensing.^56–58^

In this study, we use SLE technology to fabricate a monolithic microfluidic fused-silica probe (SLE-MFP) for nano-DESI MSI. By employing computer-aided design and manufacturing (CAD/CAM) techniques, we explored several designs of SLE-MFP. An important advancement of our approach is the substantial reduction in the laborious manual polishing and grinding steps, which streamlines the probe fabrication procedure. We describe the design and fabrication process and evaluate the performance of SLE-MFP in nano-DESI MSI experiments demonstrating the effectiveness of different probe designs in molecular imaging applications.

## 2. Methods

### 2.1 Reagents and materials

HPLC grade water and LC-MS grade methanal were purchased from Acros Organics (New Jersey). Lysophosphatidylcholine 17:1 (LPC 17:1) was purchased from Avanti Polar Lipids (Alabaster, AL). The extraction solvent used in this study was composed of 1 μM LPC 17:1 in 9 : 1 MeOH : H_2_O (v/v). Fused silica capillaries (50 μm ID, 150 μm OD) were purchased from Polymicro Technologies (Phoenix, AZ). The MinuteWeld epoxy glue was purchased from J-B Weld company (Sulphur Springs, TX). A 2000 grit silicon carbide sandpaper (STARCKE, Germany) was used to polish the SLE-MFP sampling port.

4′′ fused-silica substrates with a thickness of 1 mm were obtained from Siegert Wafer GmbH (Aachen, Germany). Potassium hydroxide solution (45%, extra pure) and MeOH (HPLC grade) were purchased from Carl Roth GmbH & Co. KG (Karlsruhe, Germany). Dioctyl sulfosuccinate sodium salt (≥99%) was purchased from Sigma-Aldrich GmbH (Taufkirchen, Germany).

### 2.2 Tissue preparation

Fresh frozen C57BL/6 mouse brains were purchased from BioIVT (Westbury, NY). Tissues were sectioned to 12 μm axially at −21 °C using a CM1850 Cryostat (Leica Microsystems, Wetzlar, Germany) and thaw mounted onto a glass microscope slide (IMEB, Inc Tek-Select Gold Series Microscope Slides, Clear Glass, Positive Charged). Mouse uterine tissue sections were provided by Dr. Xiaofei Sun from the Cincinnati Children's Hospital. Uterine tissue sections were prepared using our previously described procedures.^43^ Briefly, a uterine horn was collected on day 4 of pregnancy. The uterine tissue was sectioned to 12 μm using Leica CM 3050 cryostat and thaw-mounted onto precleaned polylysine coated microscope glass slides. All sections were stored in a −80 °C freezer prior to nano-DESI MSI analysis.

### 2.3 SLE-MFP fabrication

The SLE-MFP was designed as a virtual model using Autodesk Inventor Professional 2021 (San Rafael, CA, USA) CAD software. The design process considered the etch rate of pristine fused silica material (1 μm h^−1^) for dimensional accuracy. The CAD file was passed as a .step file format to the Alphacam 2017 R2 (Vero Software GmbH, Neu-Isenburg, Germany) CAM software. All laser-process parameters such as laser energy (230 nJ), repetition rate (1 MHz), feed rate (15.83 mm s^−1^), polarization (perpendicular to the feed rate vector), layer distance in *X*, *Y*-direction (2 μm) and *Z*-direction (7 μm) are defined during the CAM procedure. The CAM file is converted to a machine code and processed by a pulsed 400 fs IR-laser (*λ* = 1030 nm). The laser is focused through a 20× objective (LHM-20X-1064, NA = 0.40) into a 1 mm thick 4′′ fused-silica wafer mounted in the SLE device (FEMTOprint aHead P2, FEMTOprint SA, Muzzano, Switzerland). The entire automated in-volume laser processing step is completed less than 20 minutes for each MFP, allowing for the fabrication of more than 70 MFPs from the single 4′′ fused-silica substrate. The substrate is etched for 22 h in a 8 M potassium hydroxide solution at 85 °C. To minimize bubble aggregation in the monolithic microchannels, 0.02 wt% of dioctyl sulfosuccinate sodium salt diluted in MeOH is added to the etching solution. Dioctyl sulfosuccinate sodium salt has been previously used as an anionic surfactant for KOH etching.^59^ The etching process is conducted in a pulsed ultrasonic bath (2 min on, 13 min off). We found that adding the surfactant and ultrasonic agitation resulted in better detachment of bubbles during the etching process and thus improved the yield of probes. The average etching rate of the laser-modified channels is 230 μm h^−1^. In preliminary tests, the addition of the anionic surfactant to 8 mol L^−1^ KOH showed a 7.5% increase in the etching rate.

### 2.4 SLE-MFP based nano-DESI MSI

A 20–30 cm-long fused silica capillary is inserted into the fluidic inlet of the SLE-MFP and sealed with epoxy glue using the following procedure. First, the capillary is cut straight to obtain a flat end, which minimizes the dead volume. Second, the A and B components of the epoxy glue are mixed, and the adhesive is applied to the outside surface of the capillary. It is essential not to apply the adhesive too close to the end to prevent the capillary and subsequent microfluidic channel from clogging. However, any error during epoxy sealing can be easily reversed, as the adhesive can be incinerated at temperatures above 500 °C without affecting the fused silica material. Next, the capillary is inserted into the fluidic inlet of the SLE-MFP and carefully rotated to help distribute the adhesive evenly around the inner surface of the inlet to support better dispersion; additional adhesive is applied to the outer surface of the port to ensure a firm seal. The assembly was allowed to dry for at least 12 hours in a well-ventilated space. Finally, the sampling port of the SLE-MFP was slightly polished under a microscope to support a small and reliable liquid bridge. The details of the polishing procedure are discussed in the results section.

Nano-DESI MSI experiments were performed on a timsTOF Pro2 mass spectrometer (Bruker Daltonics, Bremen, Germany) with a custom-designed nano-DESI source.^60^ The SLE-MFP was placed in front of the extension tube of the MS inlet using a micropositioner (XYZ500-TIM, Quater Research and Development, Bend, OR). A glass slide with tissue samples is mounted onto an *XYZ* stage (Zaber Technologies Inc.) and positioned under the sampling port of the SLE-MFP. The *XYZ* stage is controlled by a custom-designed LabVIEW program. Two digital microscopes (Dino-Lite Digital Microscope, cat. No. AM7115MZTL) facilitate the setup and alignment process. During the MSI experiments, one of the microscopes is focused on the sampling port, while another is used to monitor the distance between the spray emitter tip and the extension tube of the MS inlet. A 1 mL glass syringe with a blunt metal needle filled with extraction solvent is connected to the SLE-MFP through the fused silica capillary. The syringe needle is connected to the instrument ground. The solvent is supplied through the capillary at a flow rate of 0.8 μL min^−1^. A liquid bridge is formed between the sampling port and the tissue sample surface. The solvent containing the extracted analytes is transferred through the spray channel to the mass spectrometer inlet, where the analytes are ionized by electrospray-like ionization. Imaging data are acquired in lines by scanning the sample under the probe at a constant scan rate and stepping between the lines. In this study, the experiments were performed in positive ionization mode. Ions were generated by applying a negative 3.5 kV potential to the mass spectrometer inlet. The drying gas temperature was 300 °C. The mass spectra were collected in the tims-off mode in the range of *m*/*z* 100–1350 with the acquisition rate of 10 Hz. The “three-point-plane” approach described in our previous study^22^ was used to control the distance between the sampling port of the SLE-MFP and sample surface. In this method, the tilt of the glass slide is defined by measuring the coordinates of three points outside of the sample. The *z*-position (height) of the *XYZ* stage was adjusted in real-time to compensate for the tilt of the sample. MS/MS spectra of the manually selected molecules were acquired by running several lines over the imaged sample with a mass selection window of 1 Da and a collision energy of 40–50 eV.

### 2.5 Data analysis

Each line scan is collected as an individual file (.d file format) using timsControl 4.0 software (Bruker, Bremen, Germany). Data processing is performed using a custom-designed Python code described in our previous study.^60^ Ion images are constructed by plotting the abundances of targeted *m*/*z* features in each mass spectrum (pixel) within the mass tolerance window of ±25 ppm as a function of the location on the tissue. Ion images are normalized either to the signal of the internal standard or total ion count (TIC). Candidate assignments of the observed *m*/*z* features are obtained by searching against LIPID MAPS (https://www.lipidmaps.org); final assignments are based on the MS/MS analysis.

## 3. Result and discussion

### 3.1 The design and optimization of the SLE-MFP

**Fig. 1:**
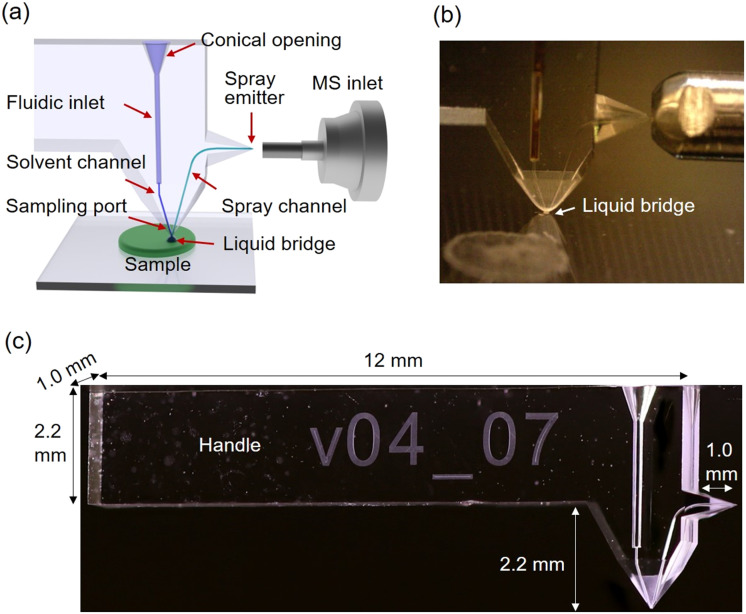
Design of the SLE-MFP for nano-DESI MSI: (a) CAD drawing sketching the microfluidic probe design and MS image setup; (b) a photograph of the SLE-MFP in front of the extension tube of a mass spectrometer source inlet; (c) a macro photograph of the monolithic SLE-MFP with the dimensions.

The design of the SLE-MFP shown in a CAD drawing (Fig. 1a) is based on the previously reported design of the iMFP.^42,43^ Specifically, the probe contains a solvent channel and a spray channel that deliver the extraction solvent to and from the sample and form a sampling port. We used a 30° angle between the channels that was reported to provide a stable liquid bridge on the sample surface.^43^ The liquid bridge with extracted analyte molecules is continuously transferred to a mass spectrometer inlet. A photograph of the probe shown in Fig. 1b illustrates its position relative to the timsTOF instrument equipped with a custom-designed extension tube described in our previous study.^60^ The dimensions of a typical SLE-MFP are shown in a photograph of the device in Fig. 1c. The probe is equipped with a long handle to simplify the mounting and positioning. The overall size of the probe is 13 mm × 4.4 mm × 1 mm. A broader 167 μm fluidic inlet is designed to connect the probe to a fused-silica capillary with an outer diameter of 150 μm that delivers the extraction solvent from a syringe pump. To simplify the insertion of the capillary, we designed a conical opening with an outer diameter of 600 μm at an angle of 20°. The sampling port and spray emitter are chamfered to ensure the stability of the extraction and ionization processes, respectively.

The microfluidic probes were manufactured using SLE, a relatively recent hybrid fabrication technique.^46^ The SLE process involves two main steps that enable precise modifications within the fused-silica material: (i) the application of femtosecond pulsed IR-laser, which generates high focal intensities leading to precisely-controlled modification within the fused-silica material. The fast and precise energy deposition by the femtosecond laser irradiation minimizes the potential for thermal damage to the fused-silica material. By moving the focal spot along a predetermined path, complex 3D structures can be inscribed into the glass volume. (ii) A wet chemical etching step using potassium hydroxide. In this step, the etching solution propagates along the inscribed structures where the etching rate is significantly higher than that of pristine fused-silica substrate. Selective etching of the modified material generates well-defined microfluidic structures inside the fused silica substrate.

In direct contrast to conventional manufacturing using photolithography, SLE offers several advantages.^49^ It is a semi-automatic process that significantly reduces processing lead time and minimizes the need for sophisticated equipment and a cleanroom facility. SLE fabrication eliminates many time-consuming and labor-intensive fabrication steps. These include thorough cleaning to reduce particle and surface contaminations, photoresist spin coating, use of optical masks, etching with toxic hydrofluoric acid, photoresist stripping, bonding of the related substrates, wafer dicing and laborious polishing of chamfers. The elimination of these manual operations also reduces scrap typically generated during critical process steps such as glass–glass wafer alignment, high-temperature bonding, and grinding/polishing of the probe.

Although isotropic etching occurs in conventional photolithography, the etching process of monolithic channels in SLE is strongly controlled by diffusion.^51^ The shape and diameter of monolithic channels fabricated using the SLE process are primarily determined by the numerical aperture of the objective used and the synergistic interplay between the laser, machine, and process-parameters. For curved channel geometries, as in the case of the spray channel (Fig. 1a) from the sampling port to the emitter, slight deviations of the developed structure from the planned design are noticeable. This is due to diffusion limitations and the resulting effects on the etching rate. In the SLE-MFP shown in Fig. 1, the spray channel is 3.2 mm in length with an average channel diameter of 53 μm ± 23 μm. The solvent channel diameter is less affected by this phenomenon since the channel is almost straight with only a 15° bend close to the interface with the fluidic inlet. As a result, the average solvent channel diameter is 50 μm ± 5 μm. The volumes of the solvent and spray channels are about 3 nL and 8 nL, respectively. The dead volume may occur between the fluidic inlet and the solvent channel. The flat end of the glass capillary which is inserted into the fluidic inlet minimizes the dead volume. The volume from the gap between the outer surface of glass capillary and the wall of fluidic inlet where no glue is applied is less than 2 nL. This volume has no effect on the performance of the SLE-MFP because it is located in the solvent channel that delivers solvent from the syringe pump. For the V-shaped probe, the same void space is present in the spray channel. We did not observe any measurable effect of this volume on the performance of the V-shaped probe likely because of the high solvent flow rate.

**Fig. 2:**
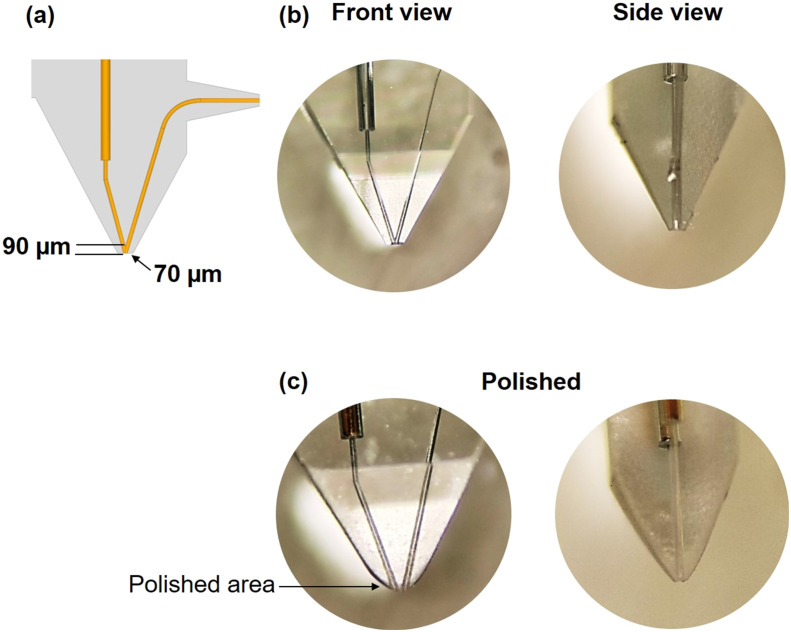
(a) Design of the sampling port of an SLE-MFP; photographs of the as-prepared SLE-MFP (b) before and (c) after polishing.

A small and stable liquid bridge is essential for nano-DESI MSI experiments. Previous studies have shown that the distance between the edge of the sampling port and the apex of the probe generated by the inner side of the channels is the key parameter that determines the size and stability of the liquid bridge.^43^ When the apex of the iMFP is located 20 μm above the edge of sampling port, it can achieve a spatial resolution of ∼30 μm and a 10-fold improvement in the experimental throughput.^43^ Furthermore, the shape of the outer part of the sampling port affects the properties of the liquid bridge. In the first design of the SLE-MFP shown in Fig. 2a, the apex of SLE-MFP is located 90 μm above the edge of the sampling port. Although the as-prepared SLE-MFP with a chamfered sampling port shown in Fig. 2b has been successfully interfaced with a mass spectrometer, we found that the liquid bridge was either too big or difficult to maintain. Based on prior experience with the iMFP, we concluded that the distance between the apex and the edge of the sampling port was too long. To improve the performance of this probe, we manually polished it using a 2000 grit silicon carbide sandpaper. Only a small amount of material was removed from the SLE-MFP to ensure its stability. The polishing process was monitored under a microscope to avoid over-polishing and took several minutes. The final shape of the sampling port after polishing is shown in Fig. 2c. The liquid bridge is much smaller after polishing as shown in Fig. S1.†

**Fig. 3:**
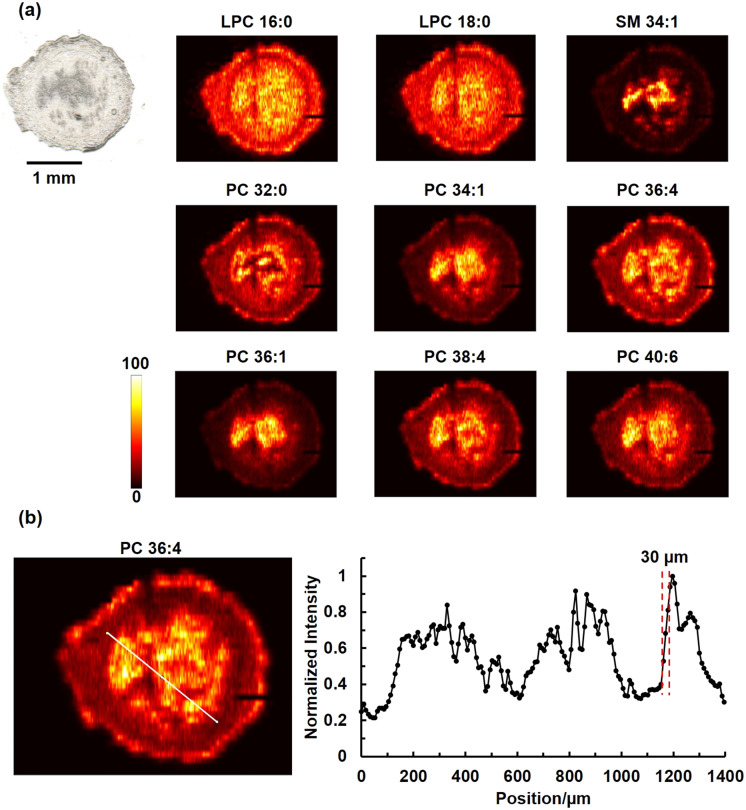
(a) Optical and representative positive mode ion images of [M + Na]^+^ ions of phospholipids in a mouse uterine tissue obtained using SLE-MFP. Scale bar: 1 mm; the intensity scale: black (low), yellow (high). The scan rate is 100 μm s^−1^, and the step between the lines is 60 μm. The acquisition rate of timsTOF is 10 Hz. Ion images are normalized to the internal standard. (b) The spatial resolution of SLE-MFP nano-DESI MSI is determined using the “20–80 rule”. Left: The ion image of [PC 36:4 + Na]^+^ with the line scan shown in white. Right: Signal profile along the line scan. The steepest gradient used to estimate the spatial resolution is shown with dashed red lines.

We used mouse uterine tissue sections to evaluate the performance of the SLE-MFP in MSI experiments. Mouse uterine tissue that contains distinct anatomical features has been used in several studies to evaluate the spatial resolution of nano-DESI MSI.^40,42,43^Fig. 3a shows an optical image of a mouse uterine tissue section and representative ion images of [M + Na]^+^ ions of selected phospholipids obtained using SLE-MFP at a scan rate of 100 μm s^−1^ and step between the lines of 60 μm. The lipids were assigned based on the accurate *m*/*z* and MS/MS data. The different cell types, including luminal epithelium (LE), glandular epithelium (GE), and stroma (S) can be well resolved. The molecular localizations obtained using SLE-MFP are consistent with previously reported results.^40,42,43^ For example, SM 34:1 is localized to the LE and GE regions, whereas the abundance of PC 32:0 is decreased in the LE region. The upper limit of the spatial resolution obtained in this experiment is 30 μm. The value was estimated using the “80–20” method,^61^ as shown in Fig. 3b. We have also demonstrated that SLE-MFP may be used for imaging in a high-throughput manner. The results of this experiment performed at a scan rate of 250 μm s^−1^ are shown in Fig. S2,† indicating the robustness of the probe at higher scan rates. These results are consistent with the reported performance of the iMFP.^42,43^

**Fig. 4:**
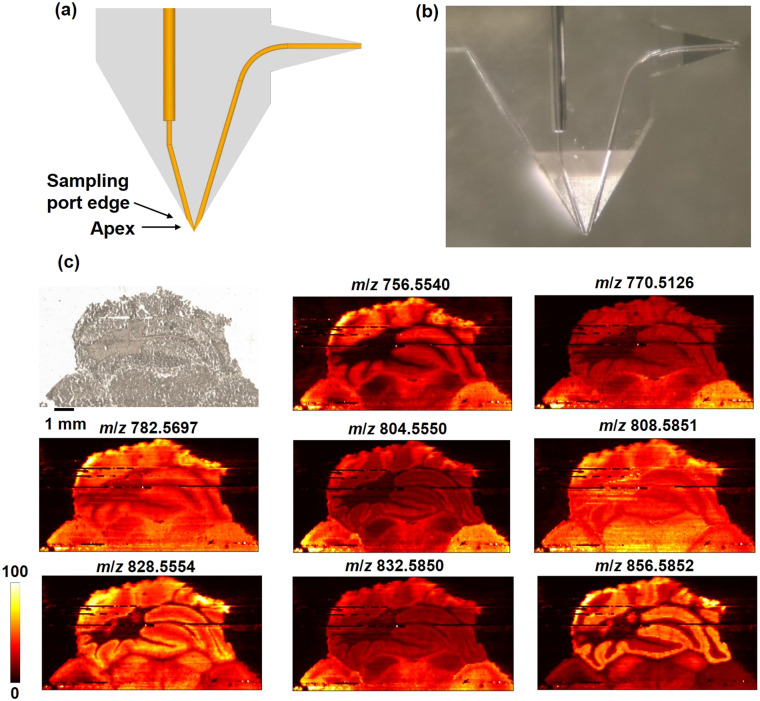
Design and evaluation of the 2nd generation SLE-MFP with a sharp sampling port for nano-DESI MSI: (a) a schematic diagram of the SLE-MFP with a sharp sampling port; (b) a photograph of the SLE-MFP with a sharp sampling port inlet; (c) optical image and representative positive ion images of molecules in mouse brain tissue obtained using the SLE-MFP. Scale bar: 1 mm; the intensity scale: black (low), yellow (high). The scan rate is 80 μm s^−1^ and the step between lines is 60 μm. The acquisition rate of MS is 10 Hz. Ion images are normalized to TIC.

Although the polishing time for SLE-MFP is much shorter than that used for the fabrication of the traditionally manufactured iMFP, it is desirable to eliminate the step of manual polishing during the fabrication of microfluidic probes for nano-DESI MSI. To further reduce the polishing time, we designed and fabricated a second generation SLE-MFP with a sharp sampling port shown in Fig. 4a and b. In this version, the edge of the probe is located above the apex, which reduces the polishing process to less than one minute. A mouse brain tissue section was used to evaluate the performance of this 2nd generation SLE-MFP and the results are shown in Fig. 4c. Aside from several lines with low signal due to the loss of the liquid bridge, this probe generates high-quality imaging data resolving fine structures of the mouse brain tissue. This result demonstrates that it is possible to eliminate the polishing step of SLE-MFP in future designs.

**Fig. 5:**
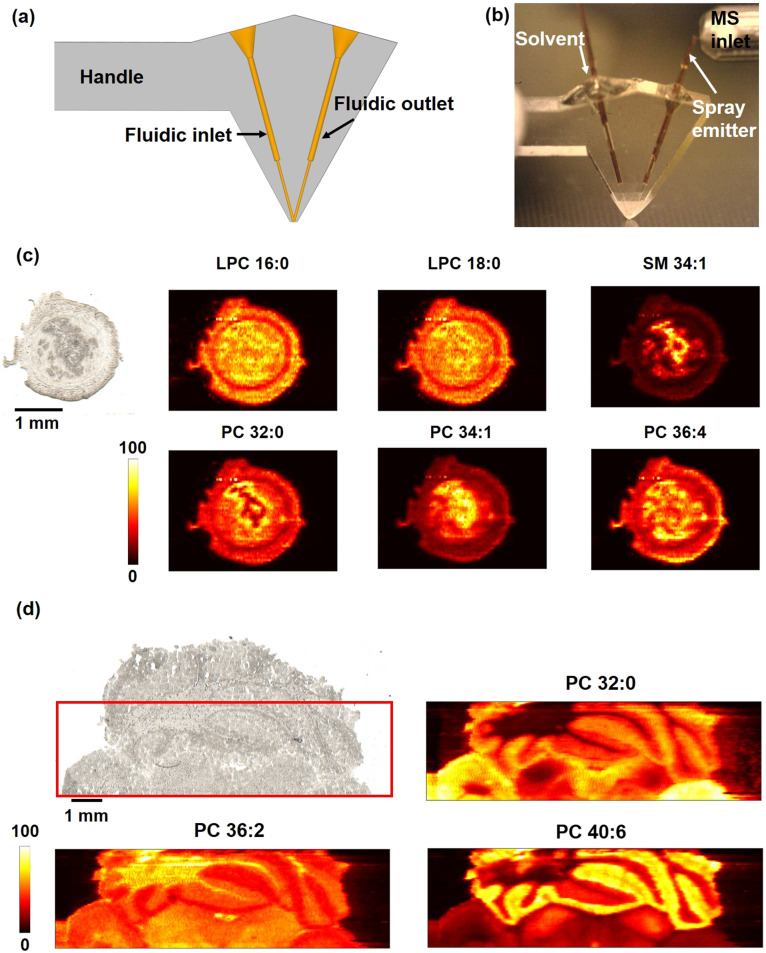
Design and evaluation of the V-shaped 3rd generation SLE-MFP for nano-DESI MSI: (a) a CAD drawing of the V-shaped SLE-MFP; (b) a photograph of the V-shaped SLE; (c) optical and representative positive mode ion images of [M + Na]^+^ ions of phospholipids in a mouse uterine tissue obtained using V-shaped SLE-MFP. Scale bar: 1 mm; the intensity scale: black (low), yellow (high). The scan rate is 50 μm s^−1^ and the step between lines is 50 μm. The ion images are normalized to the internal standard; (d) optical image and representative positive ion images of [M + Na]^+^ ions of molecules in a mouse brain tissue obtained using the V-shape SLE-MFP. The imaged sample area is marked with a red box as shown in the optical image. The scan rate is 200 μm s^−1^ and the step between lines is 50 μm. Ion images are normalized to the TIC.

Compared to the traditional manufacturing process, the SLE technology offers significantly more freedom for prototyping of the probes. New design ideas can be implemented within a very short time by designing the probes using CAD and automatically processing the designs using CAM, which facilitates remote collaboration. This quick implementation of new ideas facilitated the development of the 3rd generation SLE-MFP which combines the benefits of capillary and chip-based microfluidics using a V-shaped probe design. A CAD image of this 3rd generation SLE-MFP in which the monolithic emitter is replaced by the SLE-manufactured fluidic outlet is shown in Fig. 5a. A short-fused silica capillary is inserted and glued into the probe to serve as the spray emitter, as shown in Fig. 5b. The sampling port, in which the solvent and spray channels are precisely aligned to ensure the formation of a stable liquid bridge is the key component of this probe design. Two sockets were designed to accommodate the primary capillary and spray emitter without dead volume. The V-shaped probe is much easier to fabricate compared to the earlier generation probes and may be used to explore how the design of the emitter affects the performance of the probe. A mouse uterine tissue section was analyzed using the V-shaped SLE-MFP with a scan rate of 50 μm s^−1^ and step between the lines of 50 μm. Fig. 5c shows representative ion images obtained using the V-shaped SLE-MFP, which are consistent with the results shown in Fig. 3. The upper limit of the spatial resolution was determined to be ∼20 μm, as shown in Fig. S3.† A mouse brain tissue was used to evaluate the performance of the V-shaped SLE-MFP at a higher scan rate of 200 μm s^−1^. The results of this experiment, shown in Fig. 5d demonstrate that the V-shaped probe provides the spatial resolution sufficient to resolve the fine tissue structures of the cerebellum, including white matter, granular cell layer, and molecular layer. Additional ion images of the mouse brain tissue are provided in Fig. S4.† We note that because of the simpler design of the V-shaped SLE-MFP, it is possible to employ a higher magnification objective in the SLE machine. Thus, much smaller channel size can be realized in this configuration, which should further improve the spatial resolution of the probe.

Limitations of SLE fabrication include limited instrument availability, iterative adjustment of laser write sequence and parameters during chip development, difficulty in fabricating arbitrary channel lengths and uniform diameters due to diffusion limitations, and long etching time for monolithic devices. Despite these limitations, the fabrication process of SLE-MFP is faster, more reproducible, and more readily scalable than the conventional procedure based on photolithography, chemical etching, and bonding.^42,43^

## 4. Conclusions

We have developed a novel monolithic probe for nano-DESI MSI using SLE manufacturing, which eliminates the wafer bonding process and enables automated, scalable fabrication of the probe. In SLE, the channels inside the glass wafer are created using chemical etching of the fused-silica material that has been precisely modified using a femtosecond laser. Using this technique, we have achieved remarkable design flexibility. For example, the sampling port and ESI emitter are designed with chamfered edges, which minimizes the amount of polishing required for fine-tuning the probe. Only the sampling port of the SLE-MFP required polishing and the polishing time has been reduced to several minutes, which simplifies and streamlines the fabrication process. One of the advantages of SLE manufacturing is that it enables rapid prototyping of the microfluidic probe. This allows for quick optimization of the probe design and performance. For example, we have demonstrated that the position of the apex relative to the sampling port of the probe may be adjusted to further reduce the polishing time and improve the performance of the probe. Furthermore, we have evaluated the performance of the V-shaped SLE-MFP, in which the emitter is glued into the probe. This probe design combines the benefits of chip based and capillary based microfluidics. Our study demonstrates that SLE enables the fabrication of robust monolithic microfluidic probes, which opens up exciting opportunities for exploring different probe designs. SLE-MFP will expand the capabilities of nano-DESI MSI and make the technique more accessible to the broader scientific community.

## Conflicts of interest

There are no conflicts of interest to declare.

## Supplementary Material

LC-023-D3LC00637A-s001
