# Supplementary material for: A monolithic microfluidic probe for ambient mass spectrometry imaging of biological tissues
Source: Lab Chip. 2023 Sep 25;23(21):4664–73. doi: 10.1039/d3lc00637a (PMC10823490; doi:10.1039/d3lc00637a)
Supplement: LC-023-D3LC00637A-s001 [file LC-023-D3LC00637A-s001.pdf]

Electronic Supplementary Material (ESI) for Lab on a Chip.
This journal is © The Royal Society of Chemistry 2023

Supplementary Materials

A monolithic microfluidic probe for ambient mass spectrometry imaging of biological tissues

Li-Xue Jiang‡[1], Matthias Polack‡[2], Xiangtang Li[1], Manxi Yang[1], Detlev Belder[2],* Julia Laskin[1],*

[1]Department of Chemistry, Purdue University, West Lafayette, IN, 47907, United States

[2]Institute of Analytical Chemistry, Leipzig University, Leipzig, 04103, Germany.

E-mail: jlaskin@purdue.edu; belder@uni-leipzig.de

‡ These authors contributed equally

Table of Contents

Figure S1. Photographs of the liquid bridge generated by first generation SLE-MFP before and after polishing the sampling port………………………………………..….S2

Figure S2 Optical and representative positive ion images of the mouse uterine tissue obtained using SLE-MFP at a scan rate of 250 um/s……………….......................S3

Figure S3 The spatial resolution of V-shaped SLE-MFP nano-DESI MSI determined using the “20-80 rule”…………………………………………………………..S4

Figure S4 Optical and representative positive ion images of the mouse brain tissue obtained using V-shape SLE-MFP at a scan rate of 200 um/s……………………S5

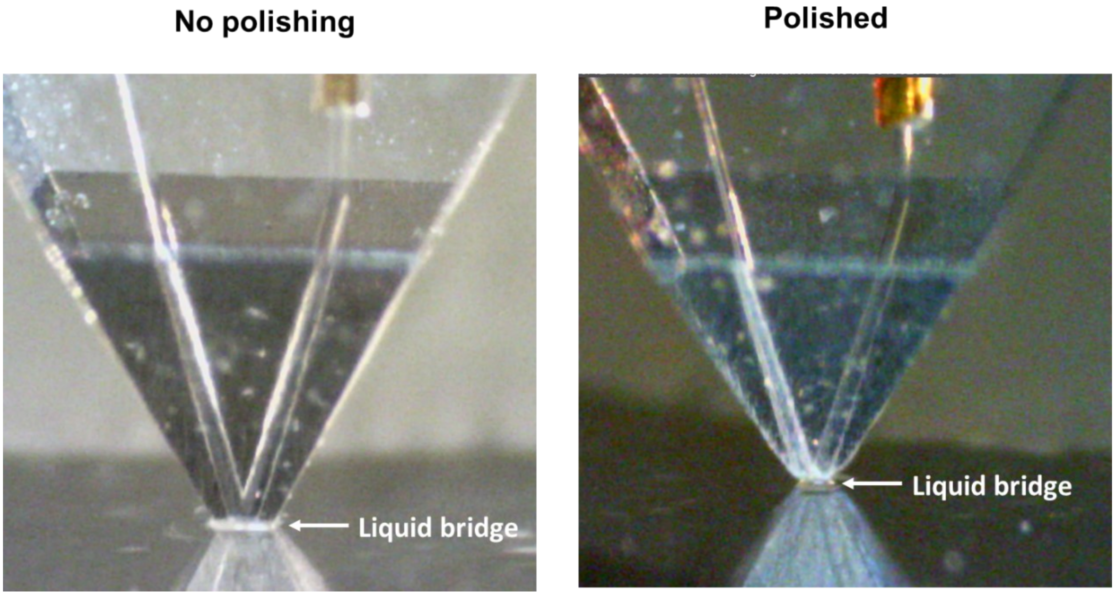

Figure S1. Photographs of the liquid bridge generated by first generation SLE-MFP before and after polishing the sampling port.

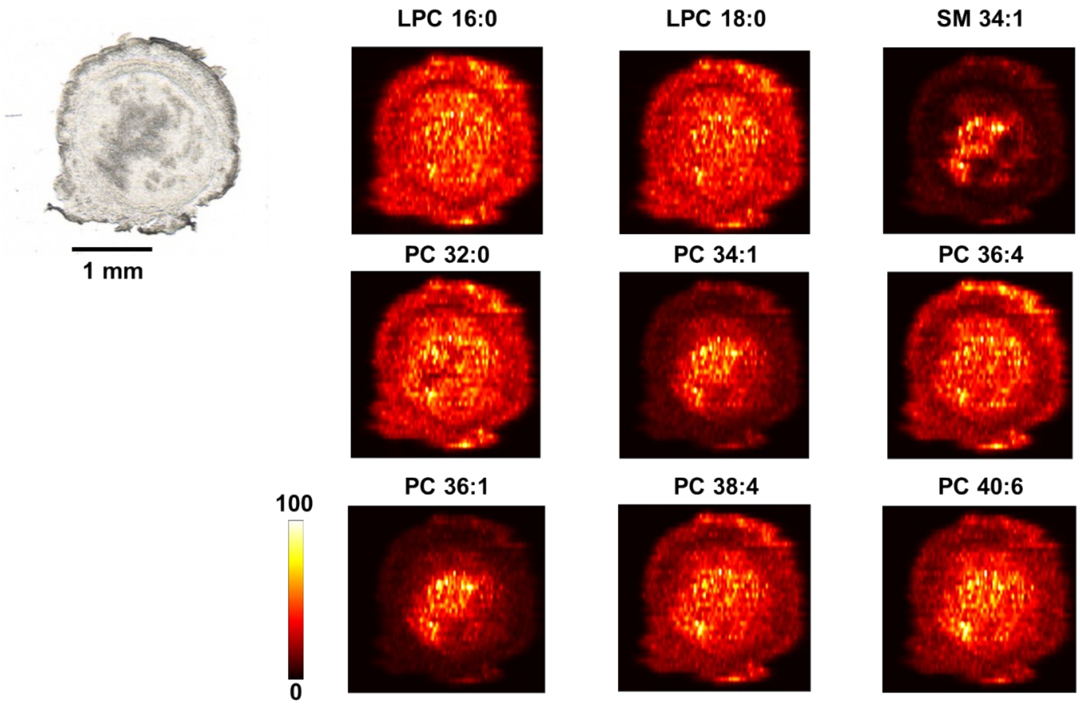

Figure S2. Optical and representative positive mode ion images of $[M+Na]^+$ ions of phospholipids in mouse uterine tissues obtained using SLE-MFP. Scale bar: 1 mm; the intensity scale: black (low), yellow (high). The scan rate is 250 µm/s and the step between lines is 60 µm. The acquisition rate of MS is 10 Hz. The ion images are normalized to the internal standard.

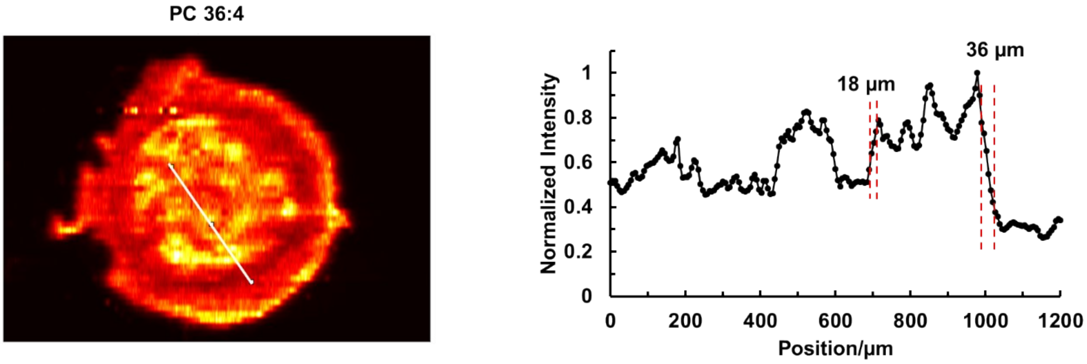

Figure S3. The spatial resolution of V-shaped SLE-MFP nano-DESI MSI determined using the "20-80 rule". Left: the ion image of $[PC\ 36{:}4 + Na]^+$ with the line scan shown in white. Right: Signal profile along the line scan. The gradient used to estimate the spatial resolution is shown with dashed red lines.

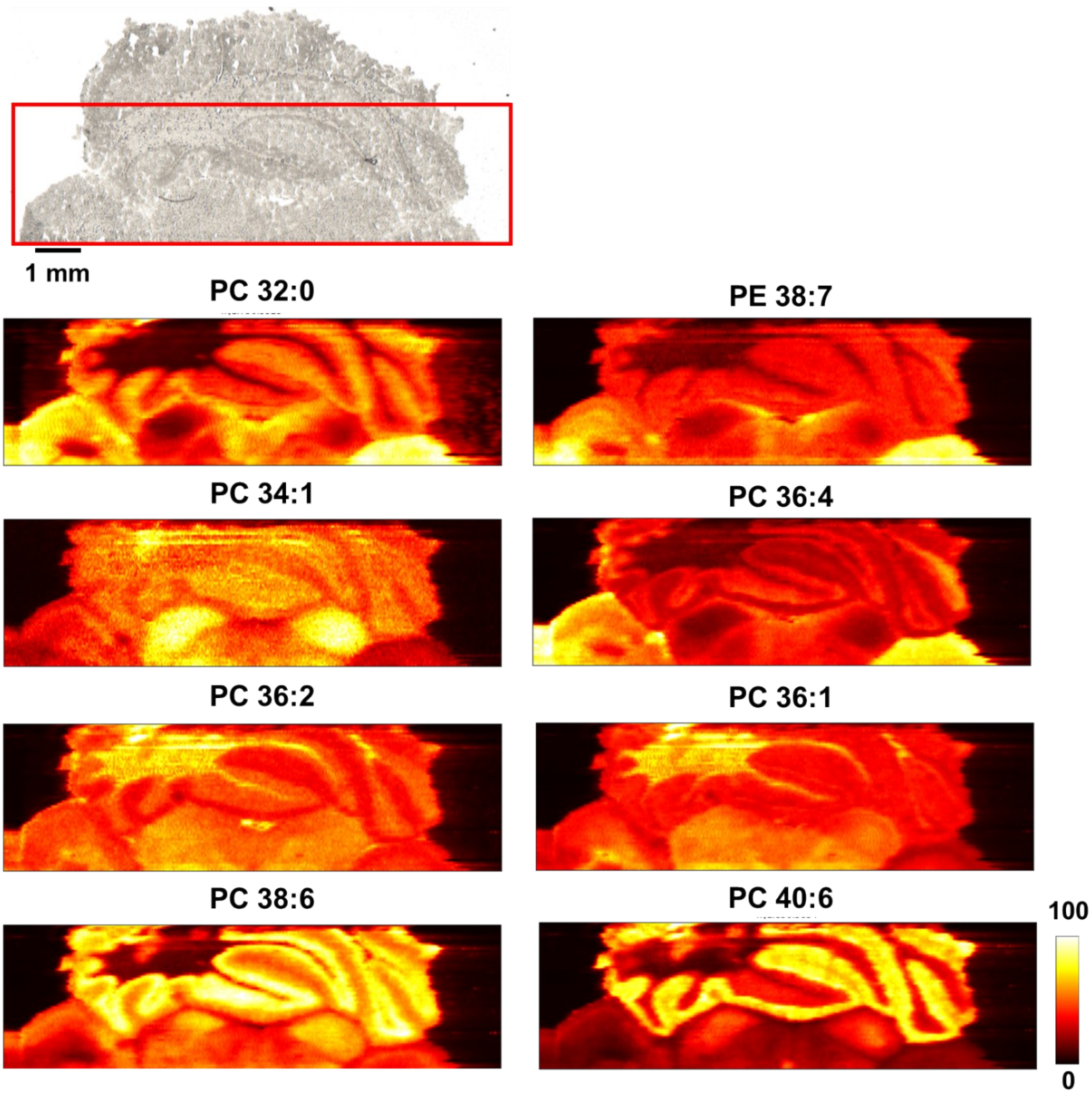

Figure S4. Optical image and representative positive ion images of $[M+Na]^+$ ions of molecules in mouse brain tissue obtained using the V-shape SLE-MFP. The imaged sample area is marked with a red box as shown in the optical image. Scale bar: 1 mm; the intensity scale: black (low), yellow (high). The scan rate is 200 µm/s and the step between lines is 50 µm. The acquisition rate of MS is 10 Hz. The ion images are normalized to the TIC.
